# Integration of intracellular telomerase monitoring by electrochemiluminescence technology and targeted cancer therapy by reactive oxygen species†
†Electronic supplementary information (ESI) available. See DOI: 10.1039/c7sc03772d


**DOI:** 10.1039/c7sc03772d

**Published:** 2017-09-25

**Authors:** Huairong Zhang, Binxiao Li, Zhaomei Sun, Hong Zhou, Shusheng Zhang

**Affiliations:** a Shandong Provincial Key Laboratory of Detection Technology for Tumor Markers , College of Chemistry and Chemical Engineering , Linyi University , Linyi 276005 , P. R. China . Email: zhouhong@lyu.edu.cn ; Email: shushzhang@126.com; b Collaborative Innovation Center of Functionalized Probes for Chemical Imaging in Universities of Shandong , Shandong Normal University , Jinan 250014 , P. R. China

## Abstract

Cancer therapies based on reactive oxygen species (ROS) have emerged as promising clinical treatments. Electrochemiluminescence (ECL) technology has also attracted considerable attention in the field of clinical diagnosis. However, studies about the integration of ECL diagnosis and ROS cancer therapy are very rare. Here we introduce a novel strategy that employs ECL technology and ROS to fill the above vacancy. Briefly, an ITO electrode was electrodeposited with polyluminol–Pt NPs composite films and modified with aptamer DNA to capture HL-60 cancer cells with high specificity. After that, mesoporous silica nanoparticles (MSNs) filled with phorbol 12-myristate 13-acetate (PMA) were closed by the telomerase primer DNA (T-primer DNA) and aptamer. After aptamer on MSN@PMA recognized and combined with the HL-60 cancer cells with high specificity, T-primer DNA on MSN@PMA could be moved away from the MSN@PMA surface after extension by telomerase in the HL-60 cancer cells and PMA was released to induce the production of ROS by the HL-60 cancer cells. After that, the polyluminol–Pt NPs composite films could react with hydrogen peroxide (a major ROS) and generate an ECL signal. Thus the intracellular telomerase activity of the HL-60 cancer cells could be detected *in situ*. Besides, ROS could induce apoptosis in the HL-60 cancer cells with high efficacy by causing oxidative damage to the lipids, protein, and DNA. Above all, the designed platform could not only detect intracellular telomerase activity instead of that of extracted telomerase, but could also kill targeted tumors by ECL technology and ROS.

## Abstract 2

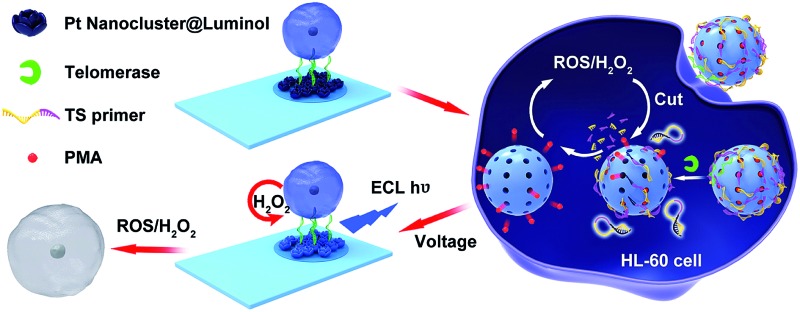
ROS and polyluminol–Pt NPs were used for intracellular telomerase detection and to induce apoptosis in HL-60 cancer cells with high efficacy.

## Introduction

Intracellular chemical detection can provide a deep understanding of cellular biological processes, and then define human healthy and diseased states.^1^ Besides, intracellular measurements could improve a variety of applications, including drug testing, toxicology, and basic cell biology.^2^ Telomerase is a ribonucleoprotein reverse transcriptase, which can extend the ends of telomeres to maintain the integrity of the process of chromosome replication. The activity of telomerase is inhibited in normal cells and is activated in cancer cells. This characteristic of telomerase makes it an important biomarker in the early diagnosis, prediction and treatment of cancer.^3^ In view of this, a high sensitivity method of intracellular telomerase activity detection is significant for disease mechanism research and clinical diagnosis.^4^

Electrochemiluminescence (ECL), as a highly sensitive detection technique, combines the advantages of both electrochemical and chemiluminescence methods and has attracted considerable attention in the field of clinical diagnosis and biomolecular detection.^5^ Luminol has been widely used in biosensor detection as a traditional ECL active material.^6^ Early reports have shown that some metal nanoparticles (Au NPs or Pt NPs) can form stable inorganic–organic complex films with polyluminol under certain conditions and that the composite films exhibit a highly sensitive analytical property for the detection of analytes.^7^ However, previously reported ECL works on telomerase detection mainly needed to extract telomerase from cells and detected the extracted telomerase activity,^8^ and the required process of extraction, preparation and storage of telomerase can probably influence the original activity of the telomerase and is expected to impact the final testing results. In view of this, an *in situ* method for detection of intracellular telomerase activity with high sensitivity is significant for studies of disease mechanism and clinical diagnosis. In this work we first used electrodeposited polyluminol–Pt NPs for *in situ* intracellular telomerase detection, which avoids the previously required extraction of telomerase from cells. Reactive oxygen species (ROS) including hydroxyl radicals (˙OH), superoxide anions (O_2_˙), hydrogen peroxide (H_2_O_2_), *etc.*, which serve dual roles in the cell life cycle, function as second messengers for several growth factors, cytokines, and signal transduction.^9^ Disrupting the balance of the ROS level could kill cells by causing oxidative damage to their lipids, protein, and DNA. Recent studies have reported a cancer treatment by ROS which has been widely adopted for clinical applications.^10^ Among ROS, H_2_O_2_ is the most stable species and has captured the interest of many researchers due to its easy assay by a variety of methods, including colorimetry, electrochemical methods, chemiluminescence and fluorescence.^11^

**Scheme 1:**
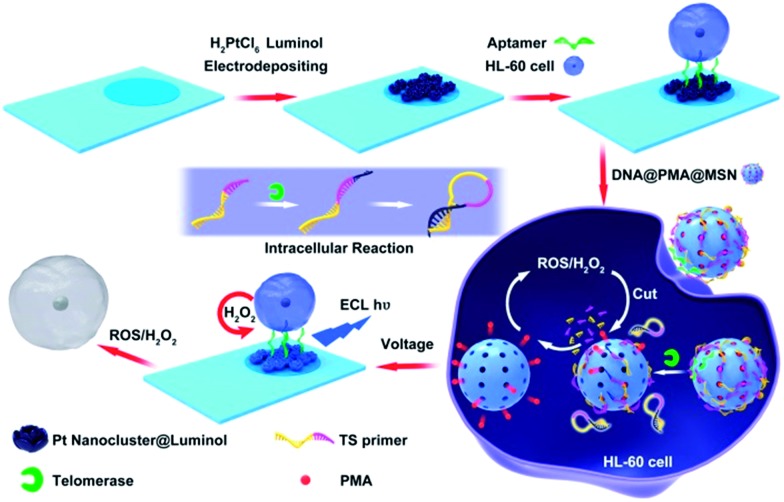
Integration of intracellular telomerase monitoring by ECL technology and targeted cancer therapy by ROS.

Therefore, based on the excellent ECL properties of polyluminol–Pt NPs composite films and the properties of ROS, a new method for monitoring intracellular telomerase activity and a targeted therapy of HL-60 cancer cells were designed (Scheme 1). Firstly, polyluminol–Pt NPs composite films modified with aptamer on the ITO electrode were used as a detection platform. Mesoporous silica nanoparticles (MSNs) filled with phorbol 12-myristate 13-acetate (PMA) and closed with T-primer DNA and aptamer DNA were used to recognize the HL-60 cancer cells, induce ECL signals and kill the HL-60 cancer cells. After MSN@PMA was endocytosed into the HL-60 cancer cells, T-primer DNA could be extended by telomerase and formed a hairpin-like DNA structure which dropped out of the MSN@PMA surface. PMA was then released and stimulated the HL-60 cancer cells to produce ROS. Furthermore, the released ROS could cause DNA oxidative cleavage reactions on the MSN surface and release more PMA which brought about the cyclic and amplified production of more ROS. The intracellular telomerase activity of the HL-60 cancer cells increased from 15 to 9000 and was monitored according to the generated ECL intensity of polyluminol–Pt NPs. Furthermore, the HL-60 cancer cell could be killed by ROS with high efficacy. Finally, the challenges of producing a simple, specific, sensitive technique for intracellular telomerase activity analysis and HL-60 cancer cell therapy by ECL technology and ROS were achieved.

## Results and discussion

**Fig. 1:**
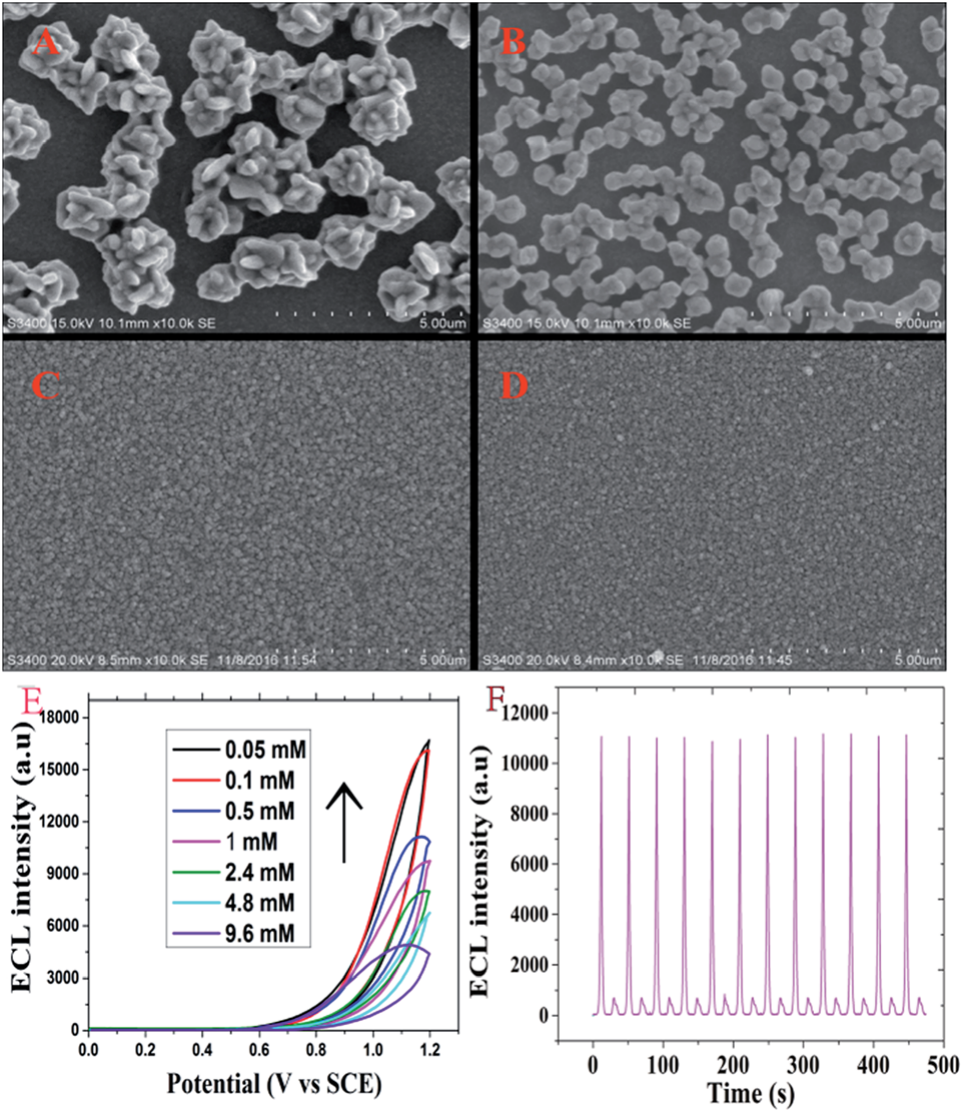
SEM images of polyluminol–Pt NPs on an ITO chip electrodeposited with different concentrations of H_2_PtCl_6_: (A) 9.6 mM; (B) 4.8 mM; (C) 2 mM and (D) 0.5 mM. The electrodepositions were performed in PBS (PH = 2) containing 0.05 M KCl at 60 °C and with an applied potential range from –0.8 V to 0.3 V for 50 cycles at 50 mV s^–1^ by cyclic voltammetry. (E) ECL curves of the polyluminol–Pt NPs composite film electroplated with different concentrations of H_2_PtCl_6_ (0.05–9.6 mM). (F) ECL emission of the polyluminol–Pt NPs composite film electroplated with 0.5 mM of H_2_PtCl_6_.

In the study, electrodeposition technology was used to build a detection platform. After the ITO chip was fabricated, H_2_PtCl_6_ and luminol could form stable polyluminol–Pt NPs composite films on the working part of the ITO chip by electrodeposition.^12^ SEM images of the polyluminol–Pt NPs electrodeposited with different concentrations of H_2_PtCl_6_ are shown in Fig. 1. These images show the particle size of the polyluminol–Pt NPs decreasing with decreasing concentrations of H_2_PtCl_6_ solution. The dispersions of the polyluminol–Pt NPs (Fig. 1A and B) with high H_2_PtCl_6_ concentrations were sparse while the dispersions of the polyluminol–Pt NPs (Fig. 1C and D) with low concentrations of H_2_PtCl_6_ tended to be smaller and more compact. The characterization above demonstrates that polyluminol–Pt NPs electrodeposited with lower concentrations of H_2_PtCl_6_ solution have larger specific surface areas and tend to be more stable. In addition, the ECL intensity of the polyluminol–Pt NPs gradually increased with decreasing concentrations of H_2_PtCl_6_ solution (Fig. 1E). This phenomenon may be attributed to the smaller polyluminol–Pt NPs having higher specific surface areas and catalytic ability. However, the polyluminol–Pt NPs composite film became unstable when the concentration of H_2_PtCl_6_ was higher than 0.5 mM. Therefore, considering its preferable stability and ECL intensity, polyluminol–Pt NPs composite film was constructed when the concentration of H_2_PtCl_6_ was 0.5 mM, and the electrodeposition was finally carried out with the potential scanning from –0.2 to 1.2 V for 50 cycles at a scan rate of 0.1 V s^–1^. As shown in Fig. 1F, the ECL measurements of the fabricated polyluminol–Pt NPs composite film showed stable and high ECL signals, which suggest that the polyluminol–Pt NPs composite film is an excellent platform to monitor intracellular telomerase activity.

**Fig. 2:**
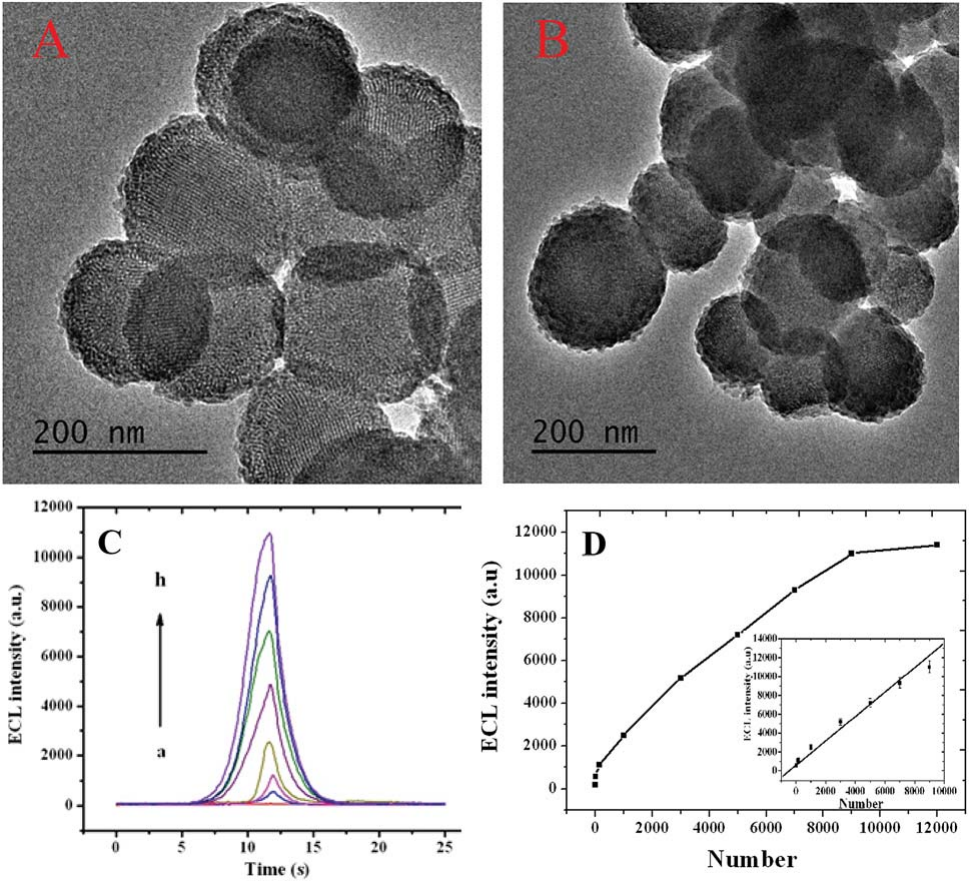
TEM image of synthesized (A) MSNs and (B) T-primer DNA-closed MSN@PMA. (C) The relationship between the ECL intensity and the number of HL-60 cancer cells, the number of HL-60 cancer cells is 0, 15, 150, 1000, 3000, 5000, 9000 and 11 000 from (a) to (h) respectively. (D) A line chart of the relationship between the ECL intensity and the number of HL-60 cancer cells; inset: the linear relationship between the ECL intensity and the number of HL-60 cancer cells, which is 15, 150, 1000, 3000, 5000 and 9000. The detection was performed in 0.1 M PBS (PH = 7.4) containing 0.1 M KCl. The applied potential ranged from –0.8 V to 1.2 V at 100 mV s^–1^.

The principle of monitoring intracellular telomerase activity based on polyluminol–Pt NPs composite films is shown in Scheme 1. MSNs, which have unique characteristics, including an easily modifiable functional surface, good biocompatibility and effective load treatment agentia, are widely used in the construction of multifunctional drug carriers.^13^ Here, MSNs were chosen and synthesized as PMA carriers due to their properties of low density and a high specific surface area.^14^ The results of dynamic light scattering (DLS) displayed in ESI Fig. S1† show an average diameter of 180 nm for the MSNs. TEM images also reflect that the synthesized MSNs show great distribution and uniform size, with an average diameter of around 180 nm (Fig. 2A). The holes on the MSNs are clearly bright which means that the mesoporous structure was successfully synthesized. However, after the MSNs were filled with PMA and closed by DNA, the holes on the MSNs become darker which means that the holes on the MSNs were successfully closed by DNA (Fig. 2B). The same result was also proven by the zeta potential which was shown in ESI Fig. S2.† On the basis of the different charges of the compounds, we can conclude that the positive charge of APTES-MSN could combine with the negatively charged T-primer DNA and finally formed the negative charge of the T-primer DNA@APTES-MSN due to electrostatic attraction. After successfully synthesizing the MSN@PMA probe, endocytosis between the HL-60 cancer cells and the MSN@PMA probe was proven by the confocal microscopic images shown in Fig. S3A.† Fluorescence imaging indicated the apparent internalization of the MSN@PMA probes by the HL-60 cancer cells. After the endocytosis reaction, a gel electrophoresis experiment was carried out to prove the feasibility of the extension of T-primer DNA by telomerase. Fig. S3B† shows an obvious 70 bp band of the extension products of T-primer DNA (lane C), which is longer than that of the T-primer DNA without extension (lane B), and the result indicates the successful extension of T-primer DNA by telomerase. Also, the T-primer DNA extension reaction by heated telomerase displays no band of extension products which means the telomerase is inactivated by heating (lane D). From the above results, MSN-PMA closed with aptamer and T-primer DNA could be endocytosed into the HL-60 cancer cells and T-primer DNA could be extended and formed the hairpin-like DNA structure in the presence of telomerase. Then, the extended DNA dropped out of the MSN probe surface and the released PMA could stimulate the HL-60 cancer cells to produce ROS. The released hydrogen peroxide generated ECL signal of the polyluminol–Pt NPs composite films and the ROS could cause apoptosis of the HL-60 cancer cells after detection. The challenge of creating a simple and specific intracellular monitoring and therapy integrated ECL technique was accomplished.

In order to get the optimized detection result, the incubation time of MSN@PMA and the HL-60 cancer cells was optimized.^15^ Fig. S4† in the ESI shows that the ECL intensities first gradually increased with increasing incubation time and tended to be stable after 90 minutes, which means that the reactions were gradually carried out to completion. Finally, 90 minutes was chosen as the optimal incubation time to make sure the action was sufficient for monitoring the intracellular telomerase activity. After incubation, MSN@PMA probe stimulated the HL-60 cancer cells to release hydrogen peroxide which generated an ECL signal with the polyluminol–Pt NPs composite film.

As shown in Fig. 2C, the ECL intensity of the polyluminol–Pt NPs composite films increased according to the increasing number of HL-60 cancer cells from 15 cells to 11 000 cells. The activity of the intracellular telomerase was then monitored. As shown in Fig. 2D, we can observe that the ECL intensities had a linear relationship with the number of HL-60 cancer cells in the range from 15 to 9000 (*R* = 0.986) with a detection limit of 15 (S/N = 3). Aptamer modified on ITO chips was used to ensure capture of the HL-60 cancer cells with specificity (Fig. S5†), and a controlled experiment indicated a quite satisfying result for the specificity. The ECL intensities of telomerase detection measured in a blank experiment, Romos cells, LO-2 cells, HopG2 cells and HL-60 cancer cells are shown in Fig. S6.† Obviously the ECL intensity increased substantially only when the HL-60 cancer cells were present. This result indicates that this approach has high specificity for detecting intracellular telomerase in HL-60 cancer cells.

**Fig. 3:**
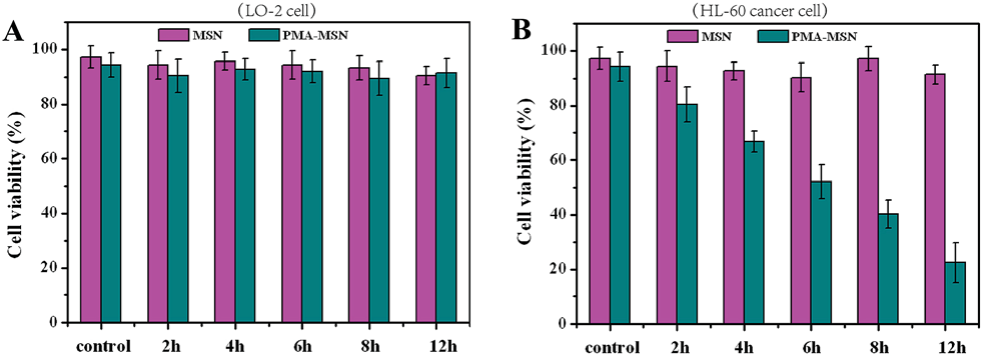
Relative viabilities of LO-2 cells (A) and HL-60 cancer cells (B) after incubation in a 100 μL culture medium containing 15 μL of pure MSNs (purple lane) and PMA@MSN probe (green lane) respectively.

We further studied the cytotoxicity of the MSN@PMA probe in the HL-60 cell line and in the non-cancerous LO-2 cell line by CCK-8 assay. According to the assay data in Fig. 3 it could be concluded that the MSNs have no cytotoxicity to either the LO-2 cells or the HL-60 cancer cells. Also, the MSN@PMA probe has no cytotoxicity to the LO-2 cells. However, after being treated with the MSN@PMA probe for 12 h, the HL-60 cells viability was less than 30% and diagnosis of HL-60 cancer cells was observable. This phenomenon is probably due to the high activity of telomerase in HL-60 cancer cells and no activity of telomerase in LO-2 cells. The active telomerase could release PMA from the MSN@PMA probe which may result in an immediate increase in the intracellular ROS level. The concept that cancer cells are more vulnerable to increased intracellular ROS levels has spurred numerous design considerations for inducing preferential cancer cell death by ROS-mediated cancer treatments.

**Fig. 4:**
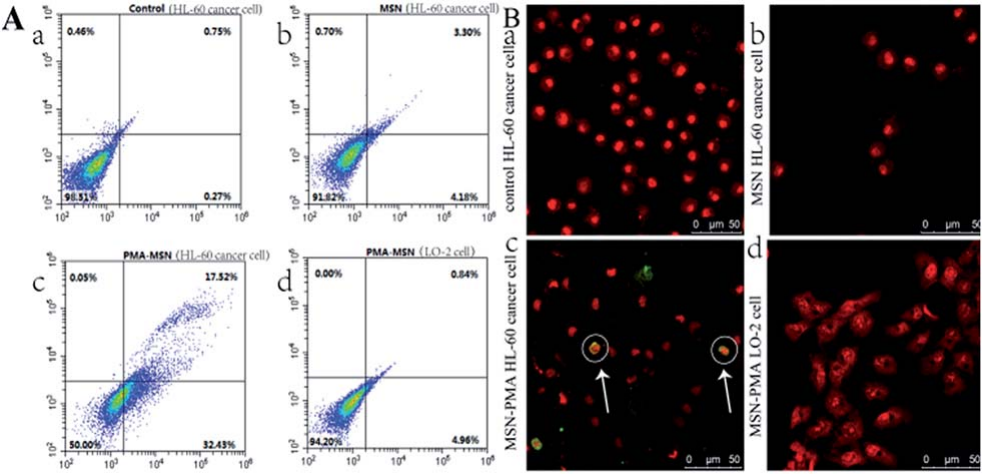
(A) Flow cytometry study of HL-60 cells (0.5 mL, 1 × 10^6^ cells per mL) after incubation with (a) PBS buffer, (b) MSNs and (c) PMA@MSN probe (1 mg mL^–1^) for 12 h and stained with the Annex V-FITC/PI apoptosis kit. (d) Flow cytometry study of LO-2 cells (0.5 mL, 1 × 10^6^ cells per mL) after incubation with PMA@MSN probe (1 mg mL^–1^) for 12 h and stained with Annex V-FITC/PI. (B) Merged confocal microscopy images of the TUNEL-FITC/PI staining of HL-60 cancer cells after treatment with (a) PBS buffer, (b) MSNs and (c) PMA@MSN probe for 12 h, respectively. (d) Merged confocal microscopy images of LO-2 cells incubated with PMA@MSN probe for 12 h and stained with TUNEL-FITC/PI.

The degree of cell membrane damage and the disruption of phosphatidylserine distribution on the cell membranes were revealed by Annexin V-FITC/PI co-staining and a flow cytometry assay, showing 31.4% of the HL-60 cancer cells in an early apoptosis stage after 12 h incubation with MSN@PMA, significantly higher than those of the control samples (Fig. 4A, and Fig. S7†, ESI). It is worth noting that the elevated ROS level may take over and trigger cell apoptosis through the subsequent ROS-mediated mechanism. TUNEL staining further confirmed that HL-60 cancer cells treated with MSN@PMA underwent apoptosis with prominent DNA fragmentation. After the phenylindole (PI) staining experiment, we found that HL-60 cancer cells treated with MSNs and LO-2 cells treated with MSNs or the MSN@PMA probe had no shrunken nuclei compared with the control samples (Fig. 4B, and Fig. S8†, ESI). However, we found that the cell nuclei significantly shrank and had shape abnormality compared with the control samples after the HL-60 cancer cells were treated with the MSN@PMA probe for 12 h (Fig. 4B (c)). This phenomenon means that MSN@PMA could kill HL-60 cancer cells and be harmless to non-cancerous cells which means that the targeted therapy of cancer cells was successfully achieved here.

## Conclusions

In this work, a new intracellular telomerase detection and cancer therapy technology using an ECL method was created utilising the special ECL properties of polyluminol–Pt NPs composite films and ROS. The ECL intensity produced by polyluminol–Pt NPs composite films on an ITO electrode was applied to detect the intracellular telomerase activity instead of that of extracted telomerase in HL-60 cells. After detection, HL-60 cancer cells could be killed by ROS which were reduced into harmless materials after therapy. Compared with previous studies, this work provides a brand new method which first studies the high sensitivity, low cost and intracellular telomerase activity detection by ECL technology, and the integration of detection and targeted therapy makes the method more meaningful for application.

## Conflicts of interest

There are no conflicts to declare.

## Supplementary Material

Supplementary information
