# Supplementary material for: Integration of intracellular telomerase monitoring by electrochemiluminescence technology and targeted cancer therapy by reactive oxygen species
Source: Chem Sci. 2017 Sep 25;8(12):8025–9. doi: 10.1039/c7sc03772d (PMC5853770; doi:10.1039/c7sc03772d)
Supplement: Supplementary file 1 [file SC-008-C7SC03772D-s001.pdf]

Electronic Supplementary Material (ESI) for Chemical Science.
This journal is © The Royal Society of Chemistry 2017

Supporting Information

Integration of intracellular telomerase monitoring by electrochemiluminescence and targeted cancer therapy by reactive oxygen species

Huairong Zhang[a], Binxiao Li[b], Zhaomei Sun[a], Hong Zhou*[a] and Shusheng Zhang*[a]

[a] *Shandong Provincial Key Laboratory of Detection Technology for Tumor Markers, College of Chemistry and Chemical Engineering, Linyi University, Linyi 276005, P.R. China,*

E-mail: zhouhong@lyu.edu.cn；　shushzhang@126.com.

[b] *Collaborative Innovation Center of Functionalized Probes for Chemical Imaging in Universities of Shandong, Shandong Normal University, Jinan 250014, P. R. China*

Experimental Procedures

Materials and reagents：

Anilinen-cetyltrimethylammoniumbromide (CTABr), Hexachloroplatinic (IV) acid hexahydrate (H_2PtCl_4), 3-aminopropyltriethoxysilane (APTES) and were purchased from Sinopharm Chemical Rengent Beijing Co,.Ltd. (China). Tetraethylorthosilicate (TEOS) was purchased from Tianjin Hengxing Chemical Rengent Co,. Ltd. (China). From the Sigma-Aldrich Inc. (USA), we obtained phorbol 12-myristate 13-acetate (PMA, 99%), glycerol, Tween 20, phenylmethylsulfonyl fluoride (PMSF), luminol, ethylene glycol bis(aminoethyl ether)-N,N,N,N tetraacetic acid (EGTA), 3-[(3-Cholamidopropyl) dimethylammonio]-1-propanesulfoni-c acid (CHAPS), and tri (2-carboxyethyl) phosphine hydrochloride (TCEP); 2-(4-Amidinophenyl)-6-indolecarbamidine dihydrochloride 4',6-Diamidino-2-phenylindole dihydrochloride DAPI. Cell Counting Kit-8 (CCK-8) was obtained from Beyotime Biotechnology (Shanghai, China). Hybridization Buffer (HB, pH 7.4) containing 10 mM Tris-HCl, 50 mM NaCl, 10 mM $MgCl_2$ and 1mM EDTA was used to disperse MSN probe. 0.1 M phosphate buffer saline (PBS, PH=7) contained K_2HPO_4 and KH_2PO_4. TUNEL-FITC; Annex V-FITC/PI apoptosis kit were purchased from Shanghai qcbio Science&Technologies co., Ltd. ITO coated glass (1.1 mm thickness, 100 X resistances) was purchased from Kaivo (Zhuhai, China). All aqueous solutions were prepared using RNAase-free water. Deoxynucleotide solution mixture (dNTPs) and all the DNA were purchased from SBS Genetech Co.,Ltd. (Shanghai, China) with the following sequences:

Aptamer DNA:

5-SH−ATCCAGAGTGACGCAGCATGCCCTAGTTACTACTACTCTTTTTAGCAAACGCCCTCGCTTTGGACACGGTGGCTTAGT

T-primer DNA:

5'-(CCC TAA)$_6$ AAT CCG TCG AGC AGA GTT-3'

Fluorescein labeled T-primer DNA:

5'-CCC TAA (CCC TAA)$_5$ AAT CCG TCG AGC AGA GTT-3' and the C was labeled with fluorescein dye carboxy fluorescein

Apparatus:

A Zeiss Supra 55 scanning electron microscope was used for examining scanning electron microscopy (SEM)

images. Transmission electron microscope (TEM) images were obtained from an H-800 microscope (Hitachi, Japan). A CHI 660B electrochemical workstation (Shanghai CH Instruments Corporation, China) was used to perform electrochemical measurements of cyclic voltammetry and ECL measurements was performed with an MPI-E multifunctional electrochemical and chemiluminescent analytical system (Remax Electronic Instrument Limited Co., Xi'an, China). Zeta potential and grading analysis were performed on Malvern Zetasizer Nano ZS90 (UK). The cell images were performed on a TCS SP8 II laser confocal microscope (Leica, Germany). CCK-8 assay was performed on a microplate reader (DG5033A, China). The gel image was captured using the WD-9413B imaging system (Liuyi, China). Flow cytometry (cytoflex, beckmancoulter, america).

The designed and fabrication of the ITO electrode

The fabrication process of the detection chip is similar to the previous report. Firstly, we cut a piece of quartz glass into small pieces (3 cm × 3 cm) and these small pieces glass were washed by boiling 2-propanol which contained 2 M KOH for 20 min, followed by rinsing thoroughly with deionized water and dried at 60 °C. After that, the degassed PDMS liquid was loaded on a square glass mold which coincided with the shape of quartz glass piece at 80 °C for 30 min to curdle. After it was cooled, PDMS was stripped from the mold, and three holes were punched at the central part of the PDMS by puncher as reservoirs. Then, designed channel structure was obtained. Afterwards, the PDMS layer was attached to the quartz glass piece surface and pressed firmly. After cleaning with ultrapure water and ethanol, the detection chip was obtained and stored for further use.

Preparation of MSN@PMA probe

The mesoporous silica nanoparticle (MSN) synthesis was according to the previous report with minor modification reference.[1] After synthesized, 1.0000 g MSN was suspended in 100 mL anhydrous ethanol inside a round-bottom flask, and an excess of APTES (1 mL) was added. After the mixture was stirred continuously for 6 h at 36 °C, it was filtered, washed with ethanol, and dried at 60 °C to obtain APTES-MSN which finally dispersed in a 2 mL EP tube containing 1 mL ultrapure water. PMA (0.05 ml, 5 μg mL^{-1}) was then added in the suspension and stirred overnight at room temperature to obtain MSN@PMA which was then centrifuged, washed thoroughly with ultrapure water, and dried in vacuum as a white powder. 1 mg of MSN@PMA was dispersed in 1 mL HB and mixed with 50 μL 10 μM T-primer DNA and aptamer DNA (100 ：1), which was stirred consumingly at 37 °C for 1 h. Then, the T-primer DNA and aptamer wrapped MSN probe was obtained, after the mixture was centrifuged, washed twice with ultrapure water and resuspended in HB (1 mg mL^{-1}). At last, the obtained MSN@PMA probe was stored at 4 °C without light for further experiment.

Preparation of electropolyluminol –Pt NPs composite films modified ITO electrode

The composite film of electropolyluminol-Pt NPs on ITO electrodes was first performed here. Briefly, the ITO electrode was cleaned thoroughly in an ultrasonic bath with ethanol, and water subsequently and dried with blowed nitrogen. After cleaning, the electrode was immersed into 5.0 mL electrolyte which contained 0.05 M phosphate balanced solution (pH = 2), 0.05 M KCl and different concentration H_2PtCl_6 solution. The experiment was performed by cyclic voltammetry and nitrogen gas was kept over the electrolyte at 60 °C. The potential was applied from -0.8 V to 0.3 V for 50 cycles at 0.05 V/s. Under these conditions, the tinct of the ITO electrode transformed from colorless into yellow, indicating that Pt NPs were deposited on the electrode successfully. Afterwards, the electrodes with Pt NPs were washed and exposed to 5 mL H_2SO_4 (0.5 M) solution containing 1 mM luminol and 20 mM aniline to obtained the polyluminol layer. And the voltage was from - 0.2 to 1.2 V for 50 cycles at 0.1 V/s. After that, polyluminol-Pt NPs composite films modified electrodes were obtained and the color of the ITO electrode transformed from yellow into stone blue. The obtained polyluminol - Pt NPs composite films modified ITO electrodes was immersed in PBS buffer solution for the further detection.

Cell Culture and Telomerase Extraction

HL-60 cells were cultured in DMEM medium containing 1% streptomycin and penicillin, 10% fetal bovine serum (FBS) and kept at 37 °C in a moist atmosphere constituted by 5% CO_2 and 95% air. About 106 HL-60 were transferred into a 1.5 mL EP tube and washed twice with 0.1 M ice-cold PBS (pH = 7.4) by centrifugalization at 2000 rpm at 4 °C for 5min. Then, the cells were dispersed in 200 µL of ice-cold CHAPS lysis buffer (containing 1 mM EGTA, 1 mM $MgCl_2$, 10 mM Tris−HCl, pH= 7.5, 10% glycerol, 0.5% CHAPS, 5 mM β-Mercaptoethanol, 0.1 mM PMSF). The mixture was kept in an ice bath for 30 min and blowed every 10 minutes with pipette. After that, the mixture results were centrifuged at 16000 rpm for 20 min at 4 °C. After centrifugation, the supernatant as cell extracts were collected for analysis immediately or frozen at − 80 °C. For the control experiment, the telomerase extract was heat-treated at 95 °C for 10 min prior to detection.

Telomerase extension reaction.

5 µL of HL-60 cancer cells telomerase extracts were added into 115 µL of telomerase extension solution containing 20 µL telomerase substrate (TS) primer (10 µM) and 95 µL of telomerase extension buffer (0.63 mM KCl, 1.5 mM $MgCl_2$, 1 mM EGTA, 0.1 mM dNTPs, 0.05% Tween 20, 20 mM Tris−HCl buffer, pH 8.3) for 1 h at 37 °C. In the control experiments, the extracts of telomerase were treated for 20 min at 95 °C before extension reaction. After that, the resulting mixture was ready to use gel electrophoresis experiments.

The aptamer capture and intracellular endocytosis study

For purpose of HL-60 cancer cells capture study, 1×10^5 cells mL^{-1} HL-60 cancer cells were incubated with aptamer modified electrode 37 °C for 1 h. After washed twice with PBS, the HL-60 cancer cells were stained with

AO/EB and imaged by confocal microscopy (Figure S9). In order to study endocytosis effect between HL-60 cancer cells and MSN@PMA, HL-60 cancer cells were incubated with 500 μL Fluorescein labeled T-primer DNA modified MSN probes (1 mg mL^{-1}) at 37 °C for 90 min. After washed twice with PBS, the HL-60 cancer cells were imaged by confocal microscopy.

Gel electrophoresis experiments

In order to demonstrate the detachment of T-primer DNA by telomerase, gel electrophoresis was performed as follows: 15% polyacrylamide gel electrophoresis (PAGE) with ethidium bromide (EB) as the fluorescent indicator was prepared by using 1 × Tris-acetate-EDTA (TAE) buffer and performed as follows: 5 μL T-primer solution (10 μM), telomerase extension product, control group product and Ladder DNA solution (10 μM) were added in 1.5 μL loading buffer respectively as indicator. The mixture was loaded onto polyacrylamide hydro gel soaked in tris-acetate-EDTA (TAE) buffer, and ran at 120 V for 40 min. After silver staining, the gel electrophoresis was visualized under UV irradiation.

Intracellular ECL measurement of telomerase

The electropolyluminol - Pt NPs modified ITO electrode was incubated with 10^{-5} M aptamer DNA solution for 12 h. After that, ITO electrode was incubated with MSN@PMA at 37 °C for 90 min. Electrochemical measurement experiments were carried out with a three-electrode system, what is more, the modified ITO electrode as a working electrode, Pt and Ag/AgCl wire was used as counter electrode and reference electrode separately. All the detection was performed under those conditions: scan voltage range: - 0.8 to 1.2 V, scan rate: 100 mV/s, PMT: 800 V.

Cytotoxicity experiments

To assess the biocompatibility of MSN probe for HL-60 cells，the CCK-8 assay was adopted. In the wells of 96-well plate, HL-60 cells (85 μL, 1.0 × 10^6 cells mL^{-1}) were incubated with 100 μL culture medium containing 15 μL of MSN and MSN probe (1mg mL^{-1}) filled with PMA respectively for different desired times. In the meantime, HL-60 cells were dispersed within 100 μL culture solution as the control group. Then, 10 μL CCK-8 (5 mg mL^{-1}) was added in each well and kept at 37 °C for 4 h in the cell incubator. Finally, microplate reader was used to measure the absorbance of each well at 450 nm. After that ,according to the equation : Cell viability (%) = (Atest/Acontrol) × 100%，the cell viability could be obtained.

Apoptosis assessment

After HL-60 cells or LO-2 cells were incubated with probe for 12h, cell apoptosis rates were determined by Annexin V-FITC /PI Apoptosis Detcetion kit and quantified by flow cytometry following the manufacturer's instructions. Briefly, cells were harvested after incubation and stained for 15 min with 5 µL AnnexinV-FITC and 10 µL PI in the dark before analyzed by flow cytometry and 10000 cells were analyzed for each experiment. The live cells could not stain for both Annexin V-FITC and PI(Annexin V-FITC−/PI−) Cells at the early stage of apoptosis are negative for PI but positive for Annexin V-FITC (Annexin V-FITC+/PI−), whereas dead cells and late-stage apoptotic cells are stained for both PI and Annexin V-FITC (Annexin V-FITC+/PI+). Cell apoptosis was also assessed by TUNEL Apoptosis Detection Kit (FITC) after the cells were treatment with probe following the manufacturer's instructions, and showed through the confocal microscopy images.

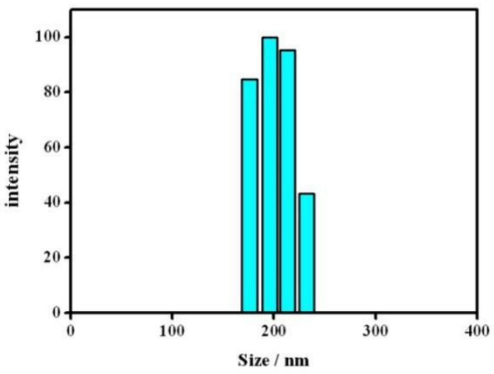

Figure S1. . Effective particle diameter of MSN probe by DLS characterization

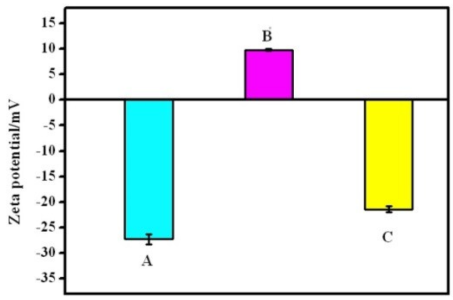

Figure S2. Zeta potential values of (A) MSN, (B) APTES-MSN, (C) T-Primer DNA-APTES@MSN (1 mg mL^{-1}) in HB buffer (PH =7.4).

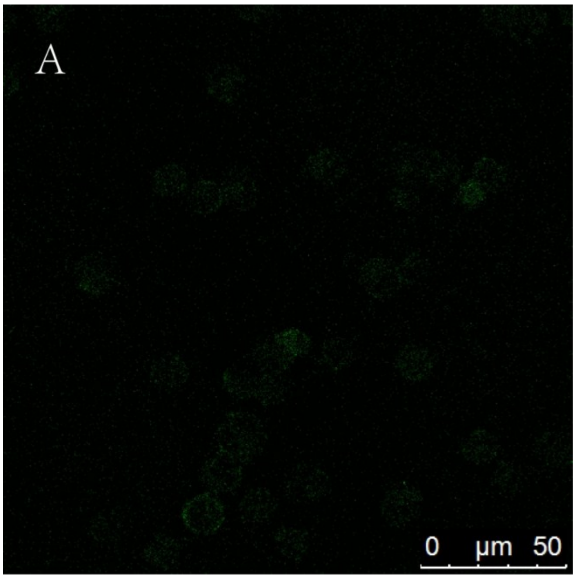

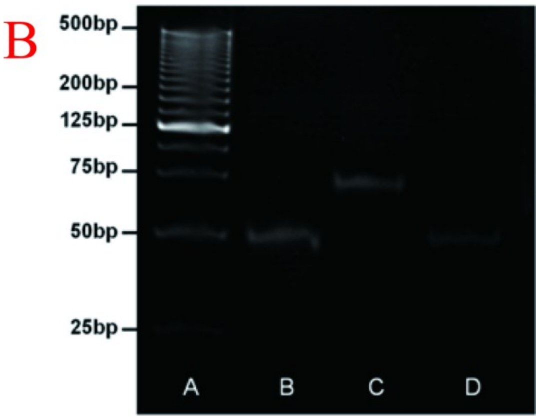

Figure S3. (A) Confocal microscopic images of (A) HL-60 cells after incubation for 90 min 500 μL incubation solution containing 80 μL MSN@PMA probe (1 mg mL-1) at 37 °C. (B) The polyacrylamide gel electrophoresis images of lane A: Ladder DNA, lane B: T-primer DNA, lane C: extension products of telomerase, lane D: extension products of heat-inactive telomerase respectively.

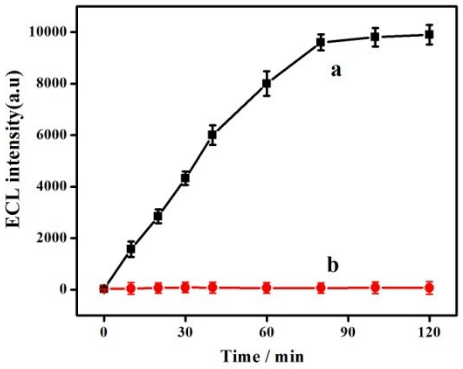

Figure S4. Plot of the ECL intensities vs. incubation time. The incubation solution (300 μL) containing 10^4 HL-60 cancer cell and in presence (a) and absence (b) of MSN@PMA probe (1mg mL^{-1}, 200 μL).

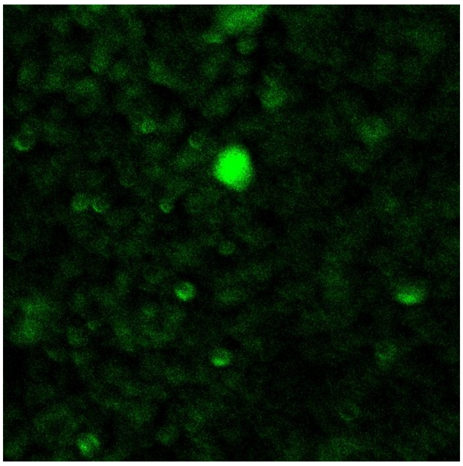

Figure S5. Confocal microscopy images of HL-60 cancer cell incubated with aptamer modified ITO chip for 1 h stained with AO/EB.

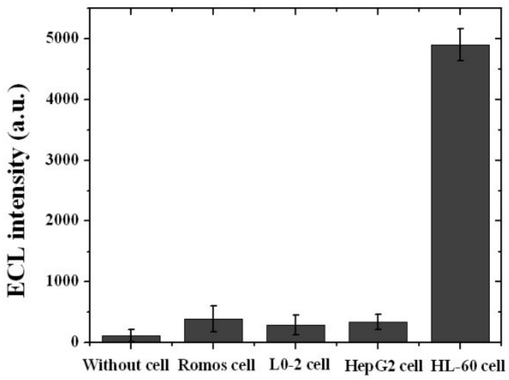

Figure S6. ECL intensities vs. different kind of cell lines. The number of cell was 3000. All the detection was performed under the following conditions, scan voltage range: − 0.8 to 1.2 V, scan rate: 100 mV/s.

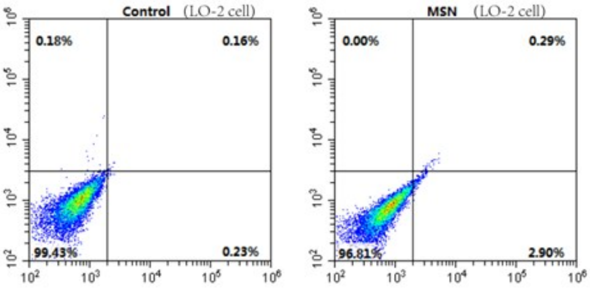

Figure S7. Flow cytometry study of LO-2 cell (0.5 mL, $1{\times}10^6$ cells mL) after incubation with 75 μL PBS buffer and MSN probe (1 mg/mL) for 12h and stained with Annex V-FITC/PI.

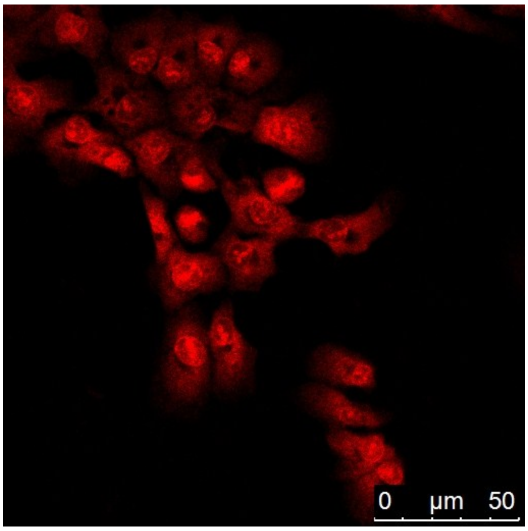

Figure S8. Merged confocal microscopy images of LO-2 cell incubated with MSN probe for 12 h and stained with TUNEL-FITC and PI.
